# An audit of ECG monitoring in patients admitted to the general adult wards at clock view hospital

**DOI:** 10.1192/bjo.2021.256

**Published:** 2021-06-18

**Authors:** Declan Hyland, Unmol Thandi, Udani Mahamithawa, Yasmine Elagamy, Mohammed Uddin

**Affiliations:** 1 Consultant Psychiatrist, Clock View Hospital, Liverpool, Mersey Care NHS Foundation Trust; 2 5th year medical undergraduate, University of Liverpool; 3 Core Trainee 1 in Psychiatry, Clock View Hospital, Liverpool, Mersey Care NHS Foundation Trust; 4 Physician Associate, Clock View Hospital, Liverpool, Mersey Care NHS Foundation Trust

## Abstract

**Aims:**

To identify whether patients admitted to the general adult inpatient wards at Clock View Hospital, an inpatient unit in Mersey Care NHS Foundation Trust, have an ECG performed following admission and whether, if this done, the ECG report is properly documented in the patient's electronic record, and whether those patients with an abnormal ECG have any further investigations requested.

**Background:**

An important risk factor for development of cardiac disease is use of psychotropic medications. Antipsychotics can increase the QTc interval.

NICE guidelines recommend that, before starting antipsychotic medication, an ECG should be offered if physical examination identifies cardiovascular risk factors, there is personal history of cardiac disease or if the individual is being admitted to hospital. The Royal College of Physicians states all patients should be assessed for cardiovascular disease, including having a routine ECG. Mersey Care's physical health policy recommends all new admissions to inpatient wards have an ECG performed within 24 hours of admission as part of their admission physical examination and investigation.

**Method:**

A sample of 60 patients discharged from the general adult wards at Clock View Hospital between 16th of July 2019 and 30th of September 2019 was obtained. An audit tool was designed and each patient's electronic record scrutinised to determine whether an ECG was performed within 24 hours of admission; in those patients who didn't, whether the reason why was recorded; and whether those patients who had an abnormal ECG were referred for further investigation. The quality of documentation of ECG reports was also analysed.

**Result:**

Age range of patients was 19–66 years. Only 31 patients had an ECG performed within 24 hours of admission. Of the remaining 29, there was documentation of why an ECG was not performed in only 16 cases. Thirteen patients had an abnormal ECG, but only three were referred for further investigation. Of the ECG reports that were analysed, only a minority met the required standard for “good”, with there being no documentation of the ECG report in one third of cases.

**Conclusion:**

There is significant room for improvement in performance of ECG monitoring and documentation of the ECG report. The importance of the ECG as part of the admission process needs to be highlighted in the induction of junior doctors. Training nursing staff on the wards to perform ECGs would reduce the likelihood of unnecessary delay to a patient having an ECG done following admission.

